# Arterial Thoracic Outlet Syndrome Treated Successfully with Totally Endoscopic First Rib Resection

**DOI:** 10.1155/2017/9350735

**Published:** 2017-08-06

**Authors:** Sofoklis Mitsos, Davide Patrini, Sara Velo, Achilleas Antonopoulos, Martin Hayward, Robert S. George, David Lawrence, Nikolaos Panagiotopoulos

**Affiliations:** Thoracic Surgery Department, University College London Hospitals (UCLH), NHS Foundation Trust, London, UK

## Abstract

Thoracic outlet syndrome (TOS) is a constellation of signs and symptoms caused by compression of the neurovascular structures in the thoracic outlet. TOS may be classified as either neurogenic TOS (NTOS) or vascular TOS: venous TOS (VTOS) or arterial TOS (ATOS), depending on the specific structure being affected. The basis for the surgical treatment of TOS is resection of the first rib, and it may be combined with scalenectomy or cervical rib resection. Herein, we describe a case of arterial thoracic outlet syndrome which was successfully treated with totally endoscopic video-assisted thoracoscopic surgery (VATS) first rib resection.

## 1. Introduction

The thoracic outlet includes three confined spaces extending from the cervical spine and mediastinum to the lower border of the pectoralis minor muscle. The three compartments are interscalene triangle, costoclavicular space, and retropectoralis minor space. The subclavian artery and the brachial plexus pass through these spaces. The subclavian vein does not cross the interscalene triangle but runs beneath the anterior scalene before joining the internal jugular vein to form the brachiocephalic vein [1]. The term “thoracic outlet syndrome” describes compression of the neurovascular structures as they exit through the cervicothoracobrachial region. These structures include the subclavian vein, the subclavian artery, and the brachial plexus and compression of any of these structures will result in venous (VTOS), arterial (ATOS), or neurogenic (NTOS) thoracic outlet syndrome, respectively [2]. The clinical presentation is highly variable with NTOS causing neurologic symptoms, being the most common in 95% of cases. Vascular involvement occurs in 5% of cases, most commonly affecting the subclavian vein (4%) followed by the subclavian artery (1%). Both conservative approach and surgical intervention are available for thoracic outlet syndrome. Whereas nonsurgical management appears to be effective in some patients, surgical treatment has been shown to offer long-term cure rates for carefully selected patients. Different invasive approaches have been described such as supraclavicular and transaxillary approaches. We report an arterial thoracic outlet syndrome case where totally endoscopic video-assisted thoracoscopic surgery (VATS) was successfully used with excellent result.

## 2. Case Presentation

**Figure 1:**
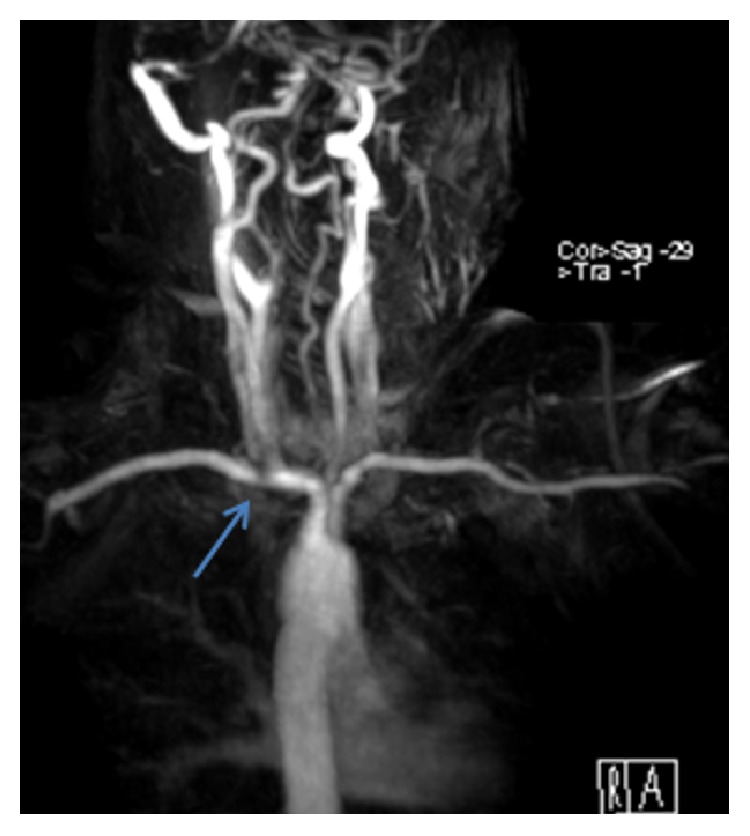
MRI showing compression (arrow) of the right subclavian artery.

A 56-year-old lady presented with severe right anterior chest pain radiating into her arm and back, with significant arm paresthesia, following a fall on her extended arm 11 months earlier. There was no evidence on the CT chest scan of any rib fractures. She had a past medical history of discectomy on her lower spine and a right carpal tunnel decompression. Diagnosis of TOS was based on clinical history, physical examination, and imaging. MRI confirmed compression in her right subclavian artery within the interscalene triangle triangle distally (Figure 1()). There was no evidence of either cervical rib involvement of the brachial plexus. These findings supported the possibility of the arterial compression to be the origin of her symptoms and surgical option was discussed with her after failure of symptoms improvement following 6 months of intense physiotherapy.

**Figure 2:**
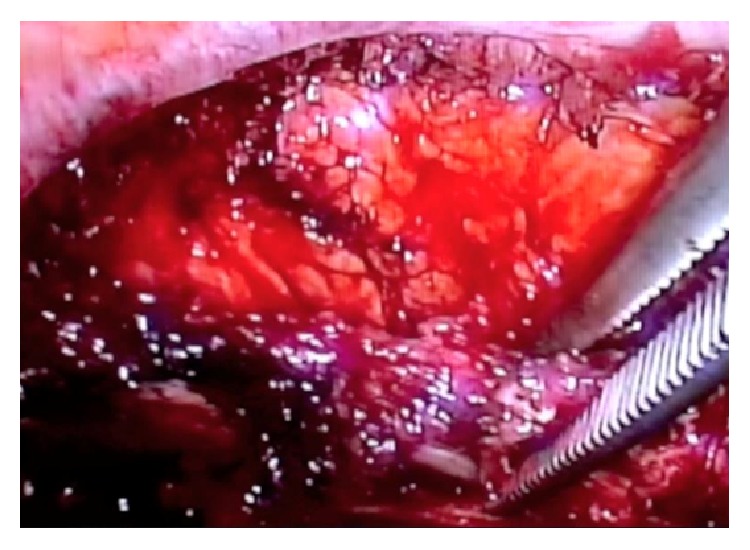
A Roberts clamp is used to retract the first rib.

**Figure 3:**
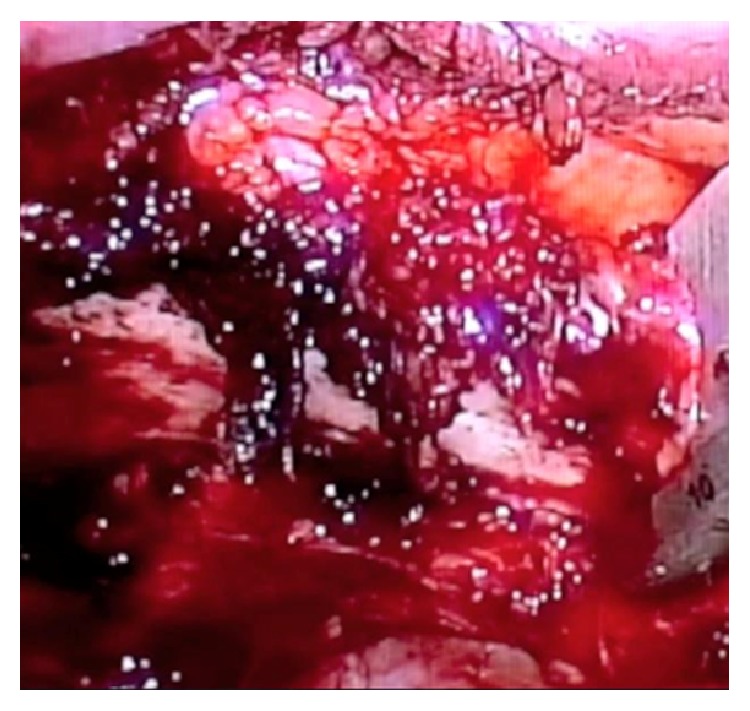
The endoscopic rib cutter is placed around the first rib.

She underwent VATS first rib resection. After intubation with double lumen tube, the patient was placed in the lateral decubitus position with the arm abducted to 90 degrees. Three standard VATS ports were utilized. Two ports were placed in the anterior third and the lateral fourth intercostal spaces, while the third scope port was placed laterally in the fifth intercostal space. While being on single lung ventilation, the apex of the right lung was gently freed from all the adhesions. The first rib was identified, and both the parietal pleura and periosteum overlying it were stripped off (Figure 2()). With blunt dissection, the rib was freed from the neurovascular bundle from above. Gentle retraction of the neurovascular bundle was performed using a peanut before passing an endoscopic rib cutter (Figure 3()). The rib was cut anteriorly near the costoclavicular junction and posteriorly at the costovertebral junction. Dissection and nibbling of both stumps were then performed. All periosteal remnants were trimmed releasing the neurovascular bundle completely.

The postoperative course was uncomplicated and patient was discharged within 24 hours following surgery. At the initial follow-up, the patient had marked improvement of her main symptoms.

## 3. Discussion

Thoracic outlet syndrome (TOS) was first described by Sir Ashley Cooper in 1821 [3]. In 1903, Bramwell recognized the first rib to be the cause of TOS and resection of the first rib for TOS was first performed in 1910.

Epidemiologic data for TOS are not widely reported. Hooper et al. suggested that this lack of demographic information is due to disagreement in the definition and diagnostic criteria for the disease [4]. The true incidence of TOS is controversial and has been reported to range from 0,3 to 8% [5]. TOS can be categorized into three distinct subtypes according to the type of compression: neurogenic (roots of the brachial plexus, 95% of cases), venous (subclavian vein, 4%-5% of cases), and arterial (subclavian artery, 1% of cases). Anatomic predispositions (cervical ribs, abnormal first ribs, alterations of the costoclavicular ligaments, anomalies of the scalene muscle, or insertion of the pectoralis minor) and extrinsic factors like trauma or chronic repetitive movements may result in TOS [6, 7].

The symptoms vary widely and include pain in the upper limb and shoulder, paresthesia, muscle weakness, claudication or pseudoangina phenomena, Paget-Schroetter syndrome or thrombosis of the subclavian vein, Raynaud's syndrome, and occasionally dermal trophic disorders, ischemia, and gangrene.

Diagnosing TOS can be challenging because of the symptoms variation. A careful history and thorough clinical examination are the most important components in establishing the diagnosis of TOS, which remains a diagnosis of exclusion and is confirmed with the reproduction of the symptoms with arm raising maneuvers, the Adson maneuver, imaging, and angiographic and neurophysiological studies [8]. Examination of patients with vascular TOS can be normal, although on occasion the distal pulse can be reduced or absent and accompanied by cyanosis or digital ischemia.

Conservative management is usually successful and consists of physical therapy, lifestyle modifications, and selective pharmacologic therapy [9]. 5% of patients, however, will have persistent symptoms and need surgical intervention.

Surgical procedures performed to relieve TOS have changed dramatically since 1861 when cervical rib resection was introduced [10, 11]. They all serve to achieve the same goal, being the decompression of the various structures that are compressed by the first rib.

Current common techniques use supraclavicular [12], transaxillary [13], or infraclavicular approach [14], each of which has its own limitations. Historically, the supraclavicular access was the most common to first rib resection until 1966, when the transaxillary approach was then described. Minimally invasive transthoracic approaches have been slow to gain popularity owing to limitations in current video-assisted thoracoscopic surgery instrumentation and technology.

Endoscopic transaxillary first rib resection has been reported [15, 16]. Strother and Margolis described a minimally invasive and successful transthoracic approach utilizing the da Vinci robotic system with excellent results and minimal morbidity [17].

In 1999, Ohtsuka et al. described a thoracoscopic approach using an endoscopic drill [18]. Loscertales et al. presented 3 cases of first rib removal by videothoracoscopy using three ports, one of which is widened for introducing periosteotomy cutters and rib shears [19]. George et al. described a new approach similar to Ohtsuka et al.'s, although it is completely endoscopic and avoids the transaxillary route, avoiding the risk of injury to the intercostobrachial cutaneous nerve [20].

VATS of thoracic outlet syndrome provides, unlike the classical approaches, an excellent view of all the bone, vascular, and nerve structures in the area. It allows for the identification of the first rib along its entire extension by simply performing the aperture on the parietal pleura. The technique allows for the removal of the first rib in its entirety from the chondrocostal union in the anterior area to the withdrawal of the costovertebral joint. Avoidance of the phrenic nerve also seems to be easier with a thoracoscopic approach as it can be traced back from its location on the pericardium and does not have to be identified in its course over the anterior scalene muscle. This approach also facilitates the section of the anterior and middle scalene muscle with perfect visualization of the artery, vein, and brachial plexus, resulting in greater safety and also improving control and preventing lesions on the sympathetic chain and the stellate ganglion.

The possible disadvantage of this approach, however, is difficult direct access to the rib from above in case of any injury to the neurovascular bundle above the rib. Another limitation is in patients with wide first rib, as the cutting area of the rib cutter is only 10 mm wide. In this case, narrowing of the rib is advised by using endoscopic bone nibbler, before passing the rib cutter around it.

The main advantages of this technique have been in the illumination and the magnified view from the VATS scope. It allows good posterior trimming of the rib and releasing of brachial plexus with a low morbidity. In addition, VATS provides a very aesthetic result which is pleasured in a female patient. The future of the thoracic outlet syndrome surgery will depend on improved techniques and specialized instruments which also help to address specific technical issues unique to the approach.
